# Radiographic Evaluation of Root Canal Fillings Accomplished by Undergraduate Dental Students

**Published:** 2015-03-18

**Authors:** Hamidreza Yavari, Mohammad Samiei, Shahriar Shahi, Zahra Borna, Amir Ardalan Abdollahi, Negar Ghiasvand, Gholamreza Shariati

**Affiliations:** a *Department of Endodontics, Dental and Periodontal Research Center, Dental School, Tabriz University of Medical Sciences, Tabriz, Iran; *; b * Department of Endodontics, Dental School, Urmia University of Medical Sciences, Urmia, Iran; *; c * Department of Operative Dentistry, Dental School, Tabriz University of Medical Sciences, Tabriz, Iran; *; d * Private practice, Tabriz, Iran*

**Keywords:** Dental Student, Radiographic Evaluation, Root Canal Fillings, Root Canal Treatment, Technical Quality

## Abstract

**Introduction:** The purpose of this study was to evaluate the radiographic quality of root canal fillings by fourth-, fifth-, and sixth-year undergraduate students at Tabriz Faculty of Dentistry between 2006 and 2012. **Methods and Materials:** A total of 1183 root canal fillings in 620 teeth were evaluated by two investigators (and in case of disagreement by a third investigator) regarding the presence or absence of under-fillings, over-fillings and perforations. For each tooth, preoperative, working and postoperative radiographs were checked. The Pearson’s chi-square test was used for statistical evaluation of the data. Inter-examiner agreement was measured by Cohen’s kappa (k) values. The level of significance was set at 0.05.** Results:** Total frequencies of over-filling, under-filling and perforation were 5.6%, 20.4% and 1.9%, respectively. There were significant differences between frequencies of over- and under-fillings (*P*<0.05). Unacceptable quality, under- and over-fillings were detected in 27.9% of 1183 evaluated canals. **Conclusion:** The technical quality of root canal therapies performed by undergraduate dental students using step-back preparation and lateral compaction techniques was unacceptable in almost one-fourth of the cases.

## Introduction

The main aim of canal obturation is to prevent re-infection of the root canal system and allow healing of periapical pathosis [1]. The quality of root canal filling (RCF) has been commonly reported as the main factor in the success of root canal treatment [2, 3]. A number of studies have assessed the quality of RCF in treatments carried out by undergraduate dental students (UDS). Epidemiological surveys have reported 10.9-91% technically acceptable RCFs performed by dental students [4-15]. This wide range is attributed to different factors considered in these studies.

Helminen *et al*. [16] showed that success or prognosis of root canal treatment depends on the technical quality of root canal filling. However, the methods used to determine the technical outcome of endodontic treatment have been generally based on radiographic evaluation [7, 9, 17-19]. 

Smith *et al*. [2] and Sjogren *et al*. [20] reported that the distance between obturation terminus and the radiographic apex, significantly affects the outcome of root canal treatment, with 87-94% of healing rates related to root fillings ending within 0-2 mm from the radiographic apex. Lower healing rates were associated with short root fillings ending more than 2 mm from the radiographic apex (68‒77.6%) and with fillings extruding outside the root apex (75‒76%) [21]. 

In addition, iatrogenic complications or procedural mishaps during root canal treatment result in imperfect RCF, and thus put the long-term consequence of treatment in jeopardy [9]. For instance, perforations are followed by infection of the periodontal ligament and the alveolar bone and subsequently compromised healing [22, 23]. Different types of root perforation (including furcation perforation, strip perforation and apical perforation) and extrusion of the root filling materials can be detected in any area along the root [24]. Furthermore, it is well known that the quality of RCF is a key factor for the prognosis of root canal therapies [17, 18, 25, 26].

Evaluation of unfavorable treatment outcomes, shows high percentages of technically unacceptable RCFs. Being aware of this inadequacy and procedural mistakes can help in providing high quality treatments and decreasing the incidence of undesirable outcomes by elevating the level of educational curriculum. As a consequence, studying the prevalence and etiology of different procedural accidents by UDSs can help the practitioner achieve an improved ending. In addition, such studies are necessary in order to assess the effectiveness of dental academic curriculum by highlighting the weak points.

Thus, the aim of this study was to evaluate the technical quality of root canal fillings using periapical radiographs of teeth treated by UDSs in Tabriz Faculty of Dentistry, Tabriz, Iran, between 2006 and 2012.

## Materials and Methods

This study was approved by the Research and Ethics Committee of Tabriz University of Medical Sciences. Records of 700 patients who had received dental treatment in the Faculty of Dentistry, Tabriz University of Medical Sciences, between 2006 and 2012 were randomly selected and investigated. Records of patients younger than 19 years of age and also the records that did not include preoperative and postoperative periapical radiographs or with clichés showing less than 2 mm of periapical region, were excluded. The cases with missing radiographs or radiographies that did not allow proper evaluation due to poor imaging or processing technique and superimposition of anatomical structures, were excluded.

Finally, documents of 620 treated cases were found eligible for evaluation. All of the endodontic treatments had been carried out by fourth, fifth, and sixth-year UDSs using K-files (Mani, Tochigi, Japan) with 0.02 taper and standard step-back technique. Canal obturation was carried out by lateral compaction technique using gutta-percha and a ZOE-based sealer. For each root-filled tooth, three clichés including preoperative, working length determination, and postoperative radiographs were inspected. 

The radiographs were mounted in a cardboard slit and interpreted in a dark room, using an illuminated Viewer box (Dentsply Rinn Corp. Elgin, IL, USA). Measurements were recorded using a transparent ruler of 0.5-mm accuracy by two Endodontists. In case of disagreement, a third investigator was asked to interpret the radiograph and a final agreement was reached.

**Table 1:** Distribution [N (%)] of inadequate fillings and iatrogenic errors in each quadrant (UR: upper right, UL: upper left, LR: lower right, LL: lower left)

	**UR**	**UL**	**LR**	**LL**
**Over-filling**	12 (1.92)	9 (1.44)	6 (0.96)	8 (1.28)
**Under-filling**	32 (5.18)	45 (7.28)	28 (4.58)	19 (3.07)
**Perforation**	3 (0.47)	1 (0.13)	4 (0.63)	3 (0.47)

Parameters used to assess radiographic quality of root fillings are listed as follows: *i)* under-filling: the root canal filling material >2 mm short of the radiographic apex; *ii)* over-filling: extrusion of the root filling material through the radiographic apex; *iii)* perforation (furcation perforation, strip perforation and apical perforation): extrusion of RCF anywhere along the root or root trunk. Radiographs were evaluated, classified and recorded. Data were revealed as percentages.

SPSS software (SPSS version 17.0, SPS SPSS, Chicago, IL, USA) was used for data processing and statistical analysis. The Pearson’s chi-square test was used for statistical evaluation of the findings. Inter-examiner agreement was measured by Cohen’s kappa (k) values among 20 of cases. The k-value was calculated as 0.64 and relatively good agreement was observed between examiners. The level of significance was set at 0.05.

## Results

Generally, the records of 350 female (56.3%) and 270 male (43.7%) patients (a total of 620 treated teeth and 1183 canals) were assessed. Of 620 observed teeth in this study, 52.9%, 14.5%, 21.6% and 11% had one, two, three and four canals, respectively.

Total rates of over-fillings, under-fillings and perforations were 5.6%, 20.4% and 1.9%, respectively. Unacceptable under- and over-filling was detected in 27.9% of canals. There were significant differences between frequencies of over- and under-fillings (*P*<0.05) with most of the failures in length control being under-filling. The distribution of inadequate fillings and iatrogenic errors in each quadrant are shown in Table 1.

The distribution of root canals with unacceptable filling according to tooth type is illustrated in Table 2. Over-filling was the most common error in first molars (3.4%). Lateral incisors and second molars (0.16%) showed the least frequency of over-filling. Under-filling was observed most frequently in first molars (8.9%) and least frequently in canines (0.64%). Perforation was reported only in first premolars and first and second molars. The first molar was the most involved tooth (Table 2). 

## Discussion

In this study a radiographic evaluation of the quality of root canal fillings was carried out among adult population referring to the Department of Endodontics, Faculty of Dentistry, Tabriz, Iran, from 2006 to 2012.

**Table 2:** Distribution [N (%)] of root canals with unacceptable fillings in different dentition groups

	**Over-filling**	**Under-filling**	**Perforation**
**Central incisor**	3 (0.48)	7 (1.13)	0 (0)
**Lateral incisor**	1 (0.16)	9 (1.45)	0 (0)
**Canine**	2 (0.32)	4 (0.64)	1 (0.15)
**First premolar**	4 (0.64)	22 (3.56)	0 (0)
**Second premolar**	1 (0.08)	21 (3.4)	7 (1.1)
**First molar**	21 (3.4)	55 (8.9)	4 (0.63)
**Second molar**	1 (0.16)	8 (1.29)	0 (0)

Many studies have considered the acceptable apical extent of the RCF within 2 mm from the radiographic apex [7, 10-13, 27]. In this study RCF with adequate length were found in 75% of teeth. Although it is difficult to compare this finding with those of other studies (because of different evaluation criteria), this percentage was noticeable compared with those in other studies with adequate length being reported to range from 54.2% to 90% [5-7, 10-14, 28, 29]. Different studies about the quality of root fillings by UDSs evaluated acceptable root filling considering several factors such as void, length, iatrogenic errors, *etc.* [6-15]. In the present study only length inadequacy and perforation were recorded.

In the present study, inadequate filling was observed in posterior teeth more than anterior teeth, with the highest percentage (4.85%) belonging to mandibular molars, similar to the study by Barrieshi-Nusair *et al. *  [10]. 

Furthermore, in several studies on nation-wide population, it is shown that molars have the highest frequency of apical periodontitis compared with other teeth [30-33]. It is apparent that in dental schools and dental practice, successful treatment of molars is difficult. Accordingly, modification of educational programs is necessary with more emphasis on the different treatment requirements for molars compared to anterior teeth.

The ratio of single-canal teeth to multiple-canal teeth in the current study was approximately 2:1. The high percentage of adequate filling in this study could be related to this relatively high proportion of single canal treatment trend. In addition, the tendency not to report problems may have been accompanied by the limitation of two-dimensional endodontic radiographic interpretation and unknown number of cases referred to the postgraduate clinic as a result of difficulties or because of technical impairment by the UDSs, which is particularly highlighted in the cases of perforation. Perforation rate in this study was very low (1.9%) in comparison with other studies, ranging from 2.7% to 11.8% [5, 9, 34, 35]. 

In this study the condition of periapical area was not considered, but in two recent studies by Moreno *et al*. [36] and Mukhaimer *et al*. [37], the periradicular status was evaluated in addition to the quality of root canal treatment; they reported that high prevalence of apical periodontitis was associated with treatments with substandard technical quality. 

One of the aims of academic courses is to improve knowledge and training through improvement of educational programs. The quality of education is the result of many factors such as time devoted to theoretical and practical teaching and training, the ratio of supervisors to students, the clinical and scientific level of teachers, whether they are specialists or not, the training aids and the assessment methods. In Tabriz Faculty of Dentistry, the ratio of supervisors to students between 2006- 2012 was approximately 1:5, which is high compared to other studies. For example in Reims (France), UK and North America this ratio was 1:11, 1:12 and 1:9, respectively [6, 38].

In the Tabriz Faculty of Dentistry, students use K-files with step-back technique. Clinical research has revealed that there is a higher incidence of procedural errors and a lower success rate of primary root canal therapies of molars with stainless steel files compared to the use of NiTi hand instruments in a continuous reaming action [12, 39]. Also, it has been shown that step-back technique, used by inexperienced students, may result in procedural mishaps which may lead to inadequate cleaning and under-filling [40-42]. Thus, teaching the crown-down technique and the use of NiTi files is recommended to improve obturation quality.

Despite the higher percentage of acceptable fillings in the present study in comparison to other studies, there should be plans to revise both preclinical and clinical curriculum of endodontics in the future to fulfill accepted standards. Moreover, further studies on factors such as homogeneity, taper of filling and other iatrogenic errors (such as ledge formation and file separation), is suggested.

## Conclusions

The radiographic quality of root canal treatments accomplished by fourth, fifth and sixth-year undergraduate students of Tabriz Faculty of Dentistry was unacceptable almost in one-fourth of cases. Thus, there is a need to improve the quality of root canal therapies performed by undergraduate students, through revision of preclinical and clinical training curriculum in Endodontic field.

## Acknowledgment

The authors thank the Research Vice Chancellor and Dental and Periodontal Research Center of Tabriz University of Medical Sciences.
